# Probable Dengue Virus Infection Among Italian Troops, East Timor, 1999–2000

**DOI:** 10.3201/eid0907.020496

**Published:** 2003-07

**Authors:** Mario Stefano Peragallo, Loredana Nicoletti, Florigio Lista, Raffaele D’Amelio, for The East Timor Dengue Study Group

**Affiliations:** * Centro Studi e Ricerche Sanità e Veterinaria Esercito, Rome, Italy; † Istituto Superiore di Sanità, Rome, Italy; ‡ Stato Maggiore della Difesa and Università “La Sapienza,” Seconda Facoltà di Medicina, Rome, Italy

**Keywords:** dengue, East Timor, military medicine, travel medicine, dispatch

## Abstract

To investigate the attack rate and risk factors for probable dengue fever, a cross-sectional study was conducted of an Italian military unit after its deployment to East Timor. Probable dengue was contracted by 16 (6.6%) of 241 army troops and caused half of all medical evacuations (12/24); no cases were detected among navy and air force personnel.

Dengue fever (DF), caused by dengue virus (DENV) serotypes 1 to 4, is an emerging public health problem in many tropical countries (*1*). Dengue hemorrhagic fever (DHF) and dengue shock syndrome (DSS), the severe manifestations of DENV infection, were first recognized in the 1950s in Southeast Asia and are today a leading cause of childhood illness and death in many tropical countries. More recently, DHF and DSS have emerged in Central and South America and in the Pacific region (*2*,*3*). DF is also recognized as an emerging health problem for international travelers (*4*,*5*) and for troops deployed to tropical countries (*6*,*7*).

In 1999, following a United Nations Security Council recommendation, the International Force for East Timor (interfet) was formed to restore peace on the island. In November 1999, Interfet troops totaled 11,000 from 17 countries. The Italian Armed Forces contributed 640 soldiers.

DF is endemic in East Timor. The peak transmission periods for DF are July–August and December–January, corresponding to the rainy months (*8*). In 1998, at least 11% of hospital inpatient deaths in East Timor were attributed to DHF (*9*). In October 1999, a localized outbreak of DF in a western district was attributed to serotype 3 (*9*) and serotype 2 was isolated in December 1999 (*10*). Serotypes 2 and 3 were also responsible for DF cases among Australian troops returning from East Timor in January–February 2000 (*11*).

During deployment, a high attack rate of febrile illness consistent with DF was reported among Italian troops. A seroepidemiologic survey was therefore conducted in February 2000 among soldiers returning home, in an attempt to determine the cause of this outbreak and to define infection rates and risk factors for infection.

## The Study

All Italian troops eligible for deployment are routinely vaccinated against diphtheria/tetanus, tetravalent meningococcal meningitis, measles/mumps/rubella, hepatitis A and B, polio (with inactivated virus), typhoid fever (orally), and yellow fever (YF). In this situation, troops were also vaccinated against Japanese encephalitis (JE) (Nakajama strain, 3 doses on days 0, 7, and 14) just before landing in East Timor.

DF prevention consisted of the use of personal protection measures against mosquitoes (repellents applied to the skin; permethrin-treated bed nets and uniforms) along with environmental mosquito control. Adulticide spraying was conducted weekly by pesticide-dispersal units but only within the campsite and in its nearest surroundings, which were also inspected daily to reduce or eliminate breeding sites of vectors.

Italian troops were deployed in East Timor from late September 1999 to mid-February 2000, and all 640 participating military personnel were eligible for inclusion in the study. Army soldiers were permanently based on the ground and operated in Dili and surrounding areas, while air force and navy personnel had only logistical tasks and their presence in Dili was episodic, since they were mainly aboard ship or based in Darwin (Australia).

A seroepidemiologic survey was conducted February 15–28, 2000, among troops returning to Italy after their 3-month period of duty in East Timor. After informed consent was obtained, peripheral blood specimens were drawn and a written questionnaire administered. The questionnaire asked for personal health data, including all symptoms experienced during deployment and information about compliance with personal protection measures. Immunization status and clinical data concerning febrile illness cases consistent with DF were obtained from standardized records kept by medical personnel. Soldiers and navy/air force personnel were studied according to their serologic status and disease status during deployment.

All specimens were screened for antibodies to dengue virus serotype 2 (DEN-2), yellow fever virus (YVF), and West Nile virus (WNV) by hemagglutination-inhibition test (HI). All serum specimens positive for DEN-2 were tested by neutralization test (NT) for DEN-2. Additionally, serum samples from participants who had experienced an acute clinical syndrome suggestive of DF were directly tested by NT for antibodies to DEN-2. Serum specimens negative for DEN-2 were then tested for neutralizing antibodies to dengue virus serotypes 1, 3, and 4 (DEN-1, DEN-3 and DEN-4).

The HI test was performed by the method of Clarke and Casals (*12*) and NT as 90% plaque reduction neutralization test (PRNT) on Vero cells. Briefly, serum specimens (twofold dilutions) and virus (10^2^ PFU) were incubated overnight at 4°C, injected onto monolayers of Vero cells, and overlaid with 1% Tragacanth gum (Sigma-Aldrich S.r.I., Milan, Italy). Seven days postinfection, cells were washed with saline and stained with 1% crystal violet in 20% ethanol (DEN-2 and DEN-3) or by immunodetection assay (DEN-1 and DEN-4) as described (*13*). Vero cells were propagated in minimum essential medium with Earle’s salts (EMEM), supplemented with nonessential amino acids, 10% fetal calf serum, 100 IE/mL of penicillin G, and 100 IE/mL of streptomycin.

The following viruses were used in the study: DEN-1 (Hawaii), DEN-2 (NGB), DEN-3 (H87), DEN-4 (H241), YF (Asibi), and WN (Bratislava). Viruses were injected into suckling mice by the intracerebral route. For NT, viral stocks were prepared as 10% brain suspension in Hank’s saline+7.5% bovine serum albumin (Sigma-Aldrich). For HI, test antigens were prepared by sucrose-acetone extraction from mouse infected brains (*12*). Monoclonal antibodies specific for DEN-1 or broadly reactive with flaviviruses were purchased from ATCC (ATCC HB112, ATCC HB47) and used as mouse ascitic fluid after injection into adult BALB/c mice.

Undetermined febrile illness was defined as an acute clinical syndrome with temperature >38.5°C, unrelated to diarrhea, malaria, or other identified infections. Suspected dengue (*14*) was defined as an undetermined febrile illness of 2–7 days’ duration, associated with two or more of the following manifestations: headache, retroorbital pain, myalgia, arthralgia, cutaneous rash. Antibody levels >1:1,280 dilutions by HI (*1*,*15*) for DEN-2 and >1:20 dilutions by NT to at least one of the four DENV serotypes were considered supportive serologic evidence of a recent dengue infection. Probable dengue (*1*,*14*) was defined as a case compatible with the clinical description of suspected DF and serologic findings supportive of a recent dengue infection.

The prevalence of undetermined febrile illness, suspected dengue, and probable dengue was compared by chi-square test among army and navy/air force personnel. Since navy and air force personnel had a limited exposure to the environment of East Timor, risk factors for probable dengue were studied only in the army contingent. A univariate analysis was first performed by Fisher exact test; each risk variable was crossed with the prevalence of probable dengue. Significance was tested at a level of α=0.05.

A multiple logistic regression model was used to determine the relationship between the outcome of probable dengue and a set of explanatory variables, and test the significance of each variable while simultaneously accounting for demographic and risk factors. The following variables were included in the model: age, rank, previous deployments in dengue-endemic areas, YF/JE vaccination, night guards, skin repellents/permethrin-treated uniforms/bed nets use, and operational versus logistic tasks. To identify a subset of variables significantly related to probable DF, the stepwise procedure was performed with the likelihood ratio test, by using at each step the p value of 0.05 as entry criterion and the p value of 0.10 as removal criterion. Univariate statistical analysis was performed with EpiInfo 6.04d software (Centers for Diseases Control and Prevention, Atlanta, GA, January, 2001)**]** and multivariate analysis by SPSS 11.0 software (SPSS Inc., Chicago, IL).

## Conclusions

Of 640 eligible participants (280 army, 93 air force, and 267 navy), 595 (93%) were included in the study: 241 army, 88 air force, and 266 navy personnel (Table 1). Serum specimens and questionnaires were obtained within 2 weeks after the troops’ return, in late February 2000.

**Table 1:** Characteristics of Italian military personnel, East Timor, 1999–2000

*Feature*	Army	Navy	Air Force	Total
No. participants	241	266	88	595
Mean age (years ± sd)	27 ± 7	28 ± 7	35 ± 7	–
Time of deployment	22/Sep/99–16/Feb/00	21/Oct/99–19/Feb/00	19/Sep/99–17/Feb/00	–
Mean duration of deployment (days ± sd)	100 ± 25	109 ± 14	41 ± 23	–
Person months	803	968	102	1,873
Presence in East Timor for ≥90 days (no. soldiers)	241	0	0	241
Episodical presence in East Timor (no. soldiers)	0	266	88	354

Some (14.5%) of the troops had previously been deployed to DF-endemic areas, primarily Somalia and Mozambique in 1992–1994. According to their immunization status versus YF and JE viruses, 100 (41.5%) of the 241 army soldiers had received vaccinations against YFV and JEV, 119 (49.4%) had been vaccinated against JEVonly, 2 (0.8%) against YF only, and 20 (8.3%) had not been vaccinated.

Undetermined febrile illness was more frequently reported (p<0.01) among army soldiers than among navy and air force personnel: 85 (35.3%) of /241 versus 13 (3.7%) of 354 , respectively. All participants with suspected dengue (n=30), with serologic results supportive of a recent dengue infection (n=27), and with a probable case of dengue n=16), belonged to the army group (Table 2).

**Table 2:** Clinical and serologic findings of recent dengue infection among Italian troops

Clinical assessment	Serologic findings of recent dengue infection*^a^*		
	No. supportive (%)	No. not supportive (%)	Total no. (%)
Undetermined febrile illness^a^	6 (22.2)	49 (22.9)	55 (22.8)
Suspected dengue^b^	16 (59.3)	14 (6.5)	30 (12.4)
Asymptomatic	5 (18.5)	151 (70.6)	156 (64.7)
Total	27 (100)	214 (100)	241 (100)

^a^All military personnel with supportive serologic findings belonged to the army contingent (N=241). Probable dengue cases are represented by the 16 soldiers with clinical manifestations compatible with DF (suspected dengue) and serologic findings supportive of a recent dengue infection. ^b^Cases are defined in the section “Materials and Methods.”

The 16 participants with probable dengue showed also a significant increase (p<0.01) in HI antibody titers to YFV (≥1:1,280 in 15/16 infected soldiers vs. 14/225 uninfected soldiers) and WNV (≥1:1,280 in 10/16 vs. 6/225). The average interval between the onset of clinical manifestations suggestive of DF and the date when blood samples were taken was 36±25 days. All 16 case-patients with probable DF had a fever >38.5°C; a saddle-back fever pattern was recorded for 5 (31.3%). Other reported symptoms included myalgia and rash in 13 (81.3%); headache in 11 (68.8%); retroorbital pain in 9 (56.3%), and adenopathy in 3 (18.8%). No patients had DHF/DSS.

The mean duration of probable DF cases was 7±3 days. Moreover, 12 of the 16 patients with probable DF were evacuated because of their clinical status. Univariate analysis of risk factors for probable DF suggested a possible protective effect of JEV vaccination and personal protection measures (Table 3). However, logistic regression analysis identified only a subset of variables significantly related to probable dengue, whose risk was higher among soldiers on duty in operational rather than logistic units, and lower among participants with regular use of bed nets (Table 4).

**Table 3:** Risk factors associated with probable dengue (univariate analysis)^a^

Demographic and *r*isk factors	No. cases of probable DF/ *n*o. soldiers exposed (%)	OR (95° C*I*)	*p* value*^b^*
Age, <26 y Yes No	10/124 (8.1) 6/117 (5.1)	1.62 (0.51 to 5.61)	0.26
Lower rank (enlisted men vs. NCOs/Officers) Yes No	9/145 (6.2) 7/96 (7.3)	0.84 (0.27 to 2.76)	0.47
Previous deployments in dengue-endemic areas Yes No	3/44 (6.8) 13/197 (6.6)	1.04 (0.18 to 4.01)	0.59
YFV vaccination Yes No	5/102 (4.9) 11/139 (7.9)	0.60 (0.16 to 1.95)	0.26
JEV vaccination Yes No	9/219 (4.1) 7/22 (31.8)	0.09 (0.03 to 0.34)	<0.01
Night guard at least once a week Yes No	6/142 (4.2) 10/99 (10.1)	0.39 (0.11 to 1.25)	0.06
Skin repellents, regular use (at least once a day) Yes No	9/209 (4.3) 7/32 (21.9)	0.16 (0.05 to 0.56)	<0.01
Use of permethrin-treated uniform Yes No	9/186 (4.8) 7/55 (12.7)	0.35 (0.11 to 1.17)	0.05
Bed nets, regular use (every night) Yes No	9/223 (4.0) 7/18 (38.9)	0.07 (0.02 to 0.26)	<0.01
On duty in operational vs. logistic units Yes No	15/179 (8.4) 1/62 (1.6)	5.55 (0.82 to 238.67)	0.05

^a^DF, dengue fever; OR, odds ratio; CI, confidence interval; NCOs, noncomissioned officers; YFV, yellow fever virus; JEV, Japanese encephalitis virus.

^b^Fisher exact test.

**Table 4:** Risk factors associated with probable dengue by multivariate analysis^a^

Risk factors	OR estimate	p value
On duty in operational vs. logistic units	11.29	<0.05
Bed nets, regular vs. nonregular use	0.04	<0.01

^a^ OR, odds ratio.

Since most of soldiers had been previously vaccinated with a flavivirus vaccine (YFV, JEV, or both), their immune response to an eventual dengue infection was expected to be a secondary (anamnestic) response, with high-titer antibodies cross-reacting with several DENV serotypes, as well as other flaviviruses (*15*). Thus, in spite of the lack of paired serum specimens, high antibody titers to DEN-2 by HI (≥1:1,280) (*1*,*16*) and to any of the four dengue virus serotypes by NT (≥1:20), after an average of 36 days from the onset of clinical manifestations compatible with dengue infection, may be considered supportive serology of a recent flavivirus infection, likely acquired during deployment.

Overall, 6.6% of army soldiers contracted probable dengue. No cases of probable DF were detected in the low-exposure group of navy and air force personnel. The high attack rate of probable dengue among the army contingent may be due to several reasons. First, DF and DHF/DSS are epidemic throughout Southeast Asia (*3*), including Indonesia (*17*); in particular, the incidence of DF markedly increased in East Timor in 1998–1999 (*18*). Secondly, the multinational deployment to East Timor took place during the rainy season (December–January), when the risk of infection is high.

Approximately 60% of troops with supportive serologic evidence of a recent dengue infection showed the clinical manifestations of classic DF, 20% had milder symptoms, and 20% were asymptomatic. This finding agrees with the U.S. troops’ experience in Somalia in 1993, where >85% of all DENV infections were symptomatic (*6*). In contrast, the overall ratio of inapparent to clinical DENV infections is quite high in persons living in disease-endemic areas, as in Indonesia, where it has been reported to be as high as 9.3 (*17*).

Performing duties outside the camp was associated with a significantly higher risk of infection, probably because vector control activities were regularly carried out within the compound. Regular use of bed nets was the only personal protection measure that significantly decreased the risk of contracting probable dengue. This finding is not new (*6*) and may have been because some of the troops were frequently on duty at night and thus slept during the day when the biting activity of dengue vectors is highest. Otherwise, the regular use of repellents (applied to the skin) and permethrin-treated uniforms seemed to decrease the risk for dengue infection, but the differences between those who did not follow these practices and those who did were not significant statistically.

DF is therefore an emerging problem for troops deployed to dengue-endemic areas, mainly because of the lack of effective preventive measures, the high attack rate, the high symptomatic/inapparent infection ratio, and the long period of being unfit for duty after the acute phase of the disease. DF may thus seriously disrupt the readiness of a military unit. Moreover, previously infected soldiers redeployed to disease-endemic areas may be at increased risk for DHF/DSS complications. Persons previously infected by a DENV serotype may be at higher risk of developing DHF/DSS, if they are subsequently infected by a different serotype. Such risks should be taken into account while planning international peace-keeping operations, and the risk of DHF among previously dengue-infected military personnel should be evaluated.

Cross-reaction by antiflavivirus antibodies induced by JEV vaccine may otherwise afford some cross-protection against DF. JEV vaccine (Nakajama strain) seems to decrease the attack rate of DHF and reduce the severity of cases for a short time (*19*). More recently, researchers have noted that prior vaccination of hamsters with a live, attenuated JEV vaccine strain (not licensed for human use) and a St. Louis encephalitis virus wild strain seems to reduce the severity of a subsequent WNV infection (*20*). Our data suggest that prior vaccination with the commercially available JEV inactivated vaccine for human use (Nakajama strain) may have some protective effect against subsequent probable DF. The decrease was, however, not significant, according to the multiple logistic regression model we used.

Our data suggest that effectiveness of routine protective measures against vector mosquitoes is far from satisfactory. A tetravalent dengue vaccine is needed to effectively reduce the risk for DF and DHF/DSS among troops deployed to tropical areas as well as to protect long-term international travelers to dengue-endemic countries.
